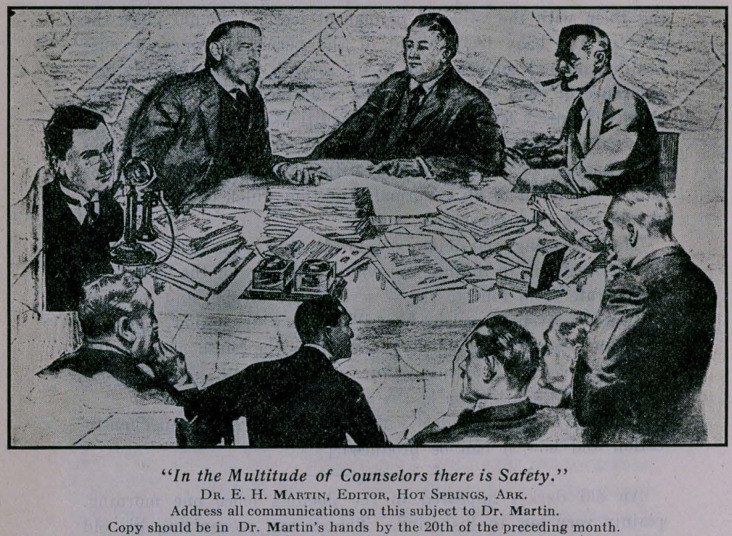# Pellagra Forum

**Published:** 1916-12

**Authors:** 


Some of our readers are young, but none are too young to have read old books, books that were written before the time of Koch and the knowledge of the cause of tuberculosis. You have all doubtless read in these old books of many lovelorn maidens who went into "a decline" and whose hectic cheeks made them more interesting. Now, if disappointment in love and ambition makes subjects more liable to succumb to tubercular bacilli, and the fact is not to be questioned, how easy it is to understand that little faults of nutrition may make one more susceptible to the unknown germ of pellagra.

Some of you may be old enough to have read the very hostile discussion which took place in the New York Academy of Medicine thirty-two years ago on the communicability of tuberculosis.

Many of you may remember frequent discussions of the question of malaria, whether it were exclusively an air-borne miasm or also a water-borne disease. I wish the dear old brother who was willing to swear to the over-ripe yellow cucumbers as a cause of malaria might read these lines.

Not more than twenty years ago we received our first knowledge of the mosquito as the intermediate host of malaria.

Since various health authorities have in the past destroyed so many hundreds of thousands of dollars worth of personal property to prevent the spread of yellow fever our P. H. and M. H. service should not feel piqued if the future discovery of the cause of pellagra should show that it had made a small mistake.

Dr. Deeks of the Canal Zone certainly can claim priority in the treatment advised by Dr. Goldberger, for both prescribe the same diet as a cure. But they approach the subject from different points of view. Deeks blames the carbohydrates for the damage done, and Goldberger blames the damage done to the lack of sufficient nitrogenous foods to balance the carbohydrates, or to some other deficiency in the diet. They are wide apart as to premises, and their points of view are exactly opposite, but the treatment advocated by each is the same. Both are right, because the diet they advocate increases the resistance of the patient to the specific organism causing the disease.

The history of the disease and our own experience shows that the germ of pellagra is a feeble organism, only thriving in the feebly resisting individual, and any diet or treatment or change of environment which increases the individual resistance enables the afflicted patient to throw off the disease.

Even alcohol with its accepted evils must be acknowledged to increase vitality when moderately used, and we see very few cases of pellagra among habitual moderate users of alcohol. In my experience only approximately 2 per cent of the cases I have seen were alcoholics, and at least 60 per cent had not had a drink or the price of a drink to spare for years.

This does not indicate that alcohol is a specific against pellagra, for the few alcoholic pellagrins I have seen have been the hardest to cure and the most apt to die. But it does indicate that those in any community who have higher vitality than the average, whether through alcohol, better living, sanitary surroundings or for any other reason, are sufficiently resistant to be immune and that pellagra only jumps on those who are down, not down in a social sense, but biologically down.

At the same time we must recognize that success in curing the disease without changing the diet or environment, as authentically observed and reported by Booth, of Drew, Mississippi, and others with cacodylate of sodium, and Allen Cox, of Helena, Arkansas, and Kelly, of Winnfield, Louisiana, and others with salvarsan, indicates that we have real specifics in the more stable of the arsenical compounds.

Dr. Dyer's results from the persistent use of quinine puzzled me for a long time. I knew that he got results, but I could not get the same. But a few months ago I found it necessary, on account of active malaria present, with fever, to delay my favorite use of salvarsan in one case of pellagra and to cure the malaria first. This was done with quinine intravenously administered.

The intravenous use of quinine popularized by Wright of Monroe, Louisiana, is one of the greatest advances in practical medical treatment offered to the profession for years. It not only gives immediate control of malaria and banishes slow fever and what we used to call congestion, but it gives us a wonderful agent in treating many cases of neuritis, especially shingles.

Not only in neuritis of malarial origin is it specific, but in many cases from other causes it is efficient, even in alcoholic neuritis.

And as pellagra organisms, with probably an intestinal habitat and a secondary infection or toxemia of the cerebro-spinal system, almost always cause toxic neuritis, giving intense pains and burning in legs and feet and at times in the upper extremities, it is not surprising that quinine given intravenously produces satisfactory results, at least on the neuritis. This proved true in the case above mentioned and caused such marked improvement that I have made the intravenous use of quinine a part of my routine treatment of pellagra.

One woman, who was making a very satisfactory recovery under the usual diet and salvarsan every ten days and soamin every other day between, got an intravenous dose of quinine every day alternately with soamin. She always welcomed the days for the intravenous doses of quinine because, as she expressed it, she "could sleep those nights without putting her burning feet out from under the cover."

An old Hindoo fable tells of three blind men who were introduced to an elephant for the first time. They groped up to examine the elephant, and as single-mindedly as most of us doctors they made their reports. One who had stumbled on the leg of the beast said that an elephant resembled a great pillar; the second, who happened to meet the elephant's trunk, declared that an elephant much resembled a great snake, and the third, who only found the tail, reported that an elephant was much like a rope. Of course they were all right, but we need not try to be blind men.

When Paul went to Athens, to spread Christianity there, he found that they knew very little about Christ, but that the Athenians were not missing anything new, for he found an altar dedicated "To the Unknown God."

The Athenian spirit is worthy of imitation when it comes to the treatment of pellagra. I use all of the treatments. When I heard Dr. Deeks of the Canal Zone read his paper in Columbia, South Carolina, in 1912, I adopted his plan of diet. When Dr. Goldberger advocated the same diet from another point of view, I stuck to it. Remembering Dr. Perdue, I often give alkalies subcutaneously while giving acids internally. Thankful to Dr. Dyer and Dr. Wright, I give quinine intravenously. Not forgetting Jelks, I give frequent doses of calomel and oil and to some cases colonic flushings. And remembering many cases treated successfully without any of the above methods I also give what I believe to be drugs specific against the causative organism, salvarsan and soamin. A number of cases treated in this way recently, using all of the above methods, have made gains of from fifteen to twenty pounds in weight in a very short time, losing all symptoms of pellagra.

Please get the idea, use all of the treatments advocated, none of them are conflicting, really, and each has a reason, probably.

E. H. M.

---

Dr. E. B. Battle has moved from New Boston to Texarkana.

---

Dr. W. H. Morgan, formerly of Marshall, is now located at Tatum, Texas.

---

"Ah," remarked the doctor, as he examined the patient's tongue and felt his pulse. "I'm glad to find you so much better this morning. Of course, you followed my prescription closely, eh?"

"Indeed, I didn't, doctor," replied the ex-invalid.

"You didn't? And why not?"

"Because if I had I should have broken my precious neck."

"Broken your neck!" gasped the man of stethoscopes in amazement. "What are you talking about?"

"I'm talking about your prescription," sighed the patient; "it blew out of the window."--*Ex.*

---

Brown—It was too bad about Dr. Smithson's death. He was only 35.

Jones—But in a way his work was finished. He had just completed his book "How to Live to Be a Hundred."—*Kansas City Star.*

## Figures and Tables

**Figure f1:**